# Mixed Berry Juice and Cellulose Fiber Have Differential Effects on Peripheral Blood Mononuclear Cell Respiration in Overweight Adults

**DOI:** 10.3390/nu15071709

**Published:** 2023-03-31

**Authors:** Patrick Solverson, George P. Albaugh, Hawi A. Debelo, Mario G. Ferruzzi, David J. Baer, Janet A. Novotny

**Affiliations:** 1 U.S. Department of Agriculture, Agricultural Research Service, Beltsville Human Nutrition Research Center, Beltsville, MD 20705, USA; 2 Department of Nutrition and Exercise Physiology, Washington State University, Spokane, WA 99202, USA; 3 Plants for Human Health Institute, North Carolina State University, Kannapolis, NC 28081, USA; 4 International Flavors and Fragrances, New Century, KS 66031, USA; 5 U.S. Department of Agriculture, Agricultural Research Service, Arkansas Children’s Nutrition Research Center, Little Rock, AR 72202, USA

**Keywords:** anthocyanins, flavonoids, cellulose, fiber, oxygraph, mitochondria, cellular respiration

## Abstract

Berries and other anthocyanin-rich foods have demonstrated anti-obesity effects in rodents and humans. However, the bioactive components of these foods and their mechanisms of action are unclear. We conducted an intervention study with overweight and obese adults to isolate the effects of different berry components on bioenergetics. Subjects consumed whole mixed berries (high anthocyanin, high fiber), pressed berry juice (high anthocyanin, low fiber), berry-flavored gelatin (low anthocyanin, low fiber), or fiber-enriched gelatin (low anthocyanin, high fiber) for one week prior to a meal challenge with the same treatment food as the pre-feed period. Peripheral blood mononuclear cells were collected 2 h after the meal challenge, and cellular respiration was assessed via high-resolution respirometry. The high-anthocyanin, low-fiber treatment (berry juice) and the low-anthocyanin, high-fiber treatment (fiber-enriched gelatin) had opposite effects on cellular respiration. In the fasted state, berry juice resulted in the highest oxygen-consumption rate (OCR), while fiber-enriched gelatin resulted in the highest OCR in the fed state. Differences were observed in multiple respiration states (basal, state 3, state 4, uncoupled), with the greatest differences being between the pressed berry juice and the fiber-enriched gelatin. Different components of berries, specifically anthocyanins/flavonoids and fiber, appear to have differential effects on cellular respiration.

## 1. Introduction

There is a large body of evidence demonstrating that oral administration of berries or anthocyanin-rich treatments demonstrate protective effects in genetic or diet-induced models of rodent obesity, as recently reviewed by Riordan and Solverson [1] and Jayarathne et al. [2]. In the majority of these studies, the addition of berry formulations or other anthocyanin-rich treatments ameliorated the obesogenic effects of a high-fat diet. Animals receiving the anthocyanin-rich treatments demonstrated lower body weights and adiposity than animals that were fed a high-fat diet without the addition of anthocyanins. These studies have shown the beneficial effects of a wide range of berry treatments, as well as other anthocyanin-rich foods. 

Recently, we translated these effects to humans through indirect calorimetry. We provided overweight/obese males a high-fat diet that included whole blackberries vs. a control food; then, we evaluated their energy expenditure and fuel utilization over a 24 h period in a room-sized indirect calorimeter. We observed that the blackberry treatment was associated with a lower respiratory quotient and increased fat oxidation [3]. 

Alterations in mitochondria could in part explain the anti-obesity effects of anthocyanins [4], and it has been suggested that whole food selection based on mitochondrial effects could be a strategy to improve body composition [5]. Mitochondria are the primary driver of cellular respiration. Mitochondrial dysfunction is described in obesity in a variety of tissues, centrally and peripherally, with implications for metabolic health and energy balance [6,7,8,9]. Mitochondrial number and function are altered in adipose and muscle tissue in both human and rodent obesity [10,11,12,13]. In vitro studies show that anthocyanins can influence a variety of pathways related to bioenergetics, including mitochondrial function, and these effects may be in part mediated by impacts on the gut microbiota and anthocyanin metabolism, as reviewed by Jayarathne et al. [2]. 

Earlier work has described the utility of characterizing cellular respiration in peripheral blood mononuclear cells (PBMCs) and supports it as a candidate tissue to study the role of a variety of disease states on energetics, as well as to determine the efficacy of promising interventions [14,15,16,17,18]. The PBMC respiration rate has been positively associated with physical strength [14], inversely associated with inflammation [14], inversely associated with fatigue [18], and inversely associated with pulmonary arterial hypertension [17] and a range of other disorders. Blood cell respiration measures are positively correlated with skeletal and cardiac muscle bioenergetics [15], suggesting that they serve as a good proxy for other tissues. By using PBMC respiration as a marker of bioenergetics, the aim of this study was to determine the effect of different berry-related interventions on cellular respiration as a possible mechanism by which berry preparations and other anthocyanin-rich foods may influence fuel use, obesity, and adiposity. Treatments were chosen to isolate the effects of different berry components (specifically anthocyanins/flavonoids and fiber) as follows: whole mixed berries, pressed berry juice, berry-flavored gelatin, and berry-flavored fiber-enriched gelatin.

## 2. Materials and Methods

### 2.1. Study Design

The feeding study was randomized, placebo-controlled, and crossed-over with four treatments, and the study was conducted at the USDA Beltsville Human Nutrition Research Center in 2018-2019, as described previously [19]. Each treatment period was 8 days in length. During the first 7 days of each treatment period, participants consumed a base diet (identical during each period) that was supplemented with one of four treatment foods: whole mixed berries (high-anthocyanin, high-fiber food); pressed mixed berry juice (high-anthocyanin, low-fiber comparator); fiber-enriched, strawberry-flavored gelatin (low-anthocyanin, high-fiber comparator); or unenriched strawberry-flavored gelatin (low-anthocyanin, low-fiber comparator). Participants were not blinded to treatment foods due to feasibility. On the morning of the eighth day, after a 12 h fast, participants provided a blood sample for the evaluation of fasted blood-based energetics, then a meal challenge was administered, followed by a blood draw at 2 h for the evaluation of post-prandial blood-based energetics. Treatment periods were separated by a two-week washout period to reduce the risk for carry-over effects. This trial was registered with ClinicalTrials.Gov (NCT03458858). 

### 2.2. Study Participants

Overweight and obese (BMI ≥ 25) male and female subjects aged 21 to 75 years old were recruited from the Washington D.C. area. Interested volunteers attended an information meeting that was presented by the Principal Investigator (J.A.N.) and provided informed consent before scheduling a screening appointment to obtain health history, anthropometrics, and urine and blood chemistries. Potential volunteers were excluded from study participation if they had any food allergies relevant to the prescribed diets; were women who had given birth in the past 12 months; were pregnant, lactating, or intended to become pregnant; had undergone weight reduction surgery; had a malabsorptive or gastrointestinal disorder, special dietary needs, or cancer diagnosis or treatment in the past 3 years; used tobacco in the preceding 6 months; used medications known to interfere with the study objectives; managed type 2 diabetes with oral medications or insulin; had elevated fasting glucose (>125 mg/dL); had abused a substance(s) in the past 12 months; or refused to give informed consent. The study protocol was reviewed and approved by Chesapeake IRB (Pro00023607), and all subjects provided written, informed consent. The feeding study was conducted at the USDA Beltsville Human Nutrition Research Center in Beltsville, MD, USA.

### 2.3. Diets and Dietary Treatment Foods

For the two weeks prior to each controlled feeding period, subjects were asked to avoid red- and purple-pigmented fruits and vegetables in order to minimize anthocyanin intake. During the week-long treatment period, subjects consumed a fully controlled diet that was devoid of anthocyanins except for the treatment foods, with a macronutrient profile of 45% of energy from fat, 40% from carbohydrate, and 15% from protein, and 14 g of fiber per day per 2000 kcal base diet. The food comprising the base diets was purchased from the following commercial food vendors: SYSCO (Jessup, MD, USA), US Foods (Rosemont, IL, USA), Giant Foods (Greenbelt, MD, USA), Shoppers Food Warehouse (College Park, MD, USA). The diets were provided based on the energy requirement for each subject so that the subjects were at weight maintenance throughout the study. Treatment foods were incorporated into the meals, were divided equally between breakfast and dinner, and were consumed under the observation of dietetic technicians. The treatment dose was scaled according to energy need, and 100 g of berries were provided per 450 kilocalories of overall dietary energy, providing a range of 400–800 g of berries per day. Control foods were matched to the berries for weight consumed, sugar composition, sugar content, and energy. Subjects were randomized to one of four treatment sequences that were generated by a statistician, and treatments were color-coded to conceal treatment identification from study staff, lab analysts, the statistician, and the investigators until after analysis was completed. 

Frozen berries were purchased in bulk and stored at −20 °C. The berry vendors were as follows: blueberries, blackberries, and raspberries from Saval Food Service (Elkridge, MD, USA); cranberries and strawberries from US Foods (Rosemont, IL, USA). The treatment foods were matched for sugar composition, total sugar content, and energy. The proximate and fiber (soluble and insoluble) analyses of the treatment foods were performed by Covance Laboratories, Inc. (Madison, WI, USA). The primary treatment food was whole mixed frozen berries, which consisted of equal parts by weight of blackberries, blueberries, cranberries, strawberries, and raspberries. Three comparator foods were designed to isolate the effects of anthocyanins, fiber, or a combination of the two and were as follows: (1) pressed berry juice, which was pressed from the same lot of berries as the primary berry treatment and filtered through cheese cloth to remove particulate (high anthocyanin, low fiber); (2) fiber-enriched, sugar-sweetened, gelatin-containing cellulose to match the fiber content and type of the whole berries (low anthocyanin, high fiber); and (3) unenriched, sugar-sweetened gelatin (low anthocyanin, low fiber) (Table 1). The cellulose source (Vital Nutrients, Middletown, CT, USA) included fiber from mixed plant species. 

### 2.4. Identification of Phenolics in Berries and Berry Juice via LC-MS/MS

Phenolic compounds were extracted from berry treatments that were adapted from methods as described previously [20,21]. Briefly, samples were defatted by extraction with 5 mL hexane 3 times. The hexane layers were discarded, and residual hexane was removed by flushing the samples with nitrogen. Phenolic extraction was then accomplished by using an extraction solution of 80% methanol, 20% water, 2% formic acid that was added to dried powder, followed by shaking and sonication for 15 min. After centrifugation, the supernatant was removed, and the residual sample was subjected to re-extraction twice more for a total of three extractions. Combined extracts were dried under vacuum (Labconco RapidVap (Kansas City, MO, USA) and reconstituted in 2% formic acid in water prior to solid-phase extraction. Samples were resolubilized with 2% formic acid in water and purified via solid-phase extraction (SPE) by using Oasis HLB 1 cc extraction cartridges (Waters, Milford, MA, USA) that were activated via sequentially rinsing with methanol and water. Extracts were loaded onto each cartridge and rinsed with 2% formic acid in water. Phenolics were then eluted with 2% formic acid in methanol. Samples were dried again under vacuum and solubilized in 200 μL of 2% formic acid in HPLC-grade water for analysis.

Extracts were analyzed for phenolic compounds on a Waters UPLC Acquity H Class system (Milford, MA, USA) with a QDa and a PDA detector. Separation was achieved with a BEH C18 column (1.7 µm, 2.1 mm × 100 mm) with a flow rate of 0.5 mL/min. The mobile phase gradient that was used was 0.1% formic acid in acetonitrile (solvent A) and 2% formic acid in water (solvent B) as follows: 0 min, 100% B; 1.0 min, 94% B; 4.0 min, 91% B; 6.0 min, 87% B; 9.0 min, 65% B; 9.8 min, 100% B; 14.5 min, 100% B. 

Phenolic standards (chlorogenic acid, ferulic acid, gallic acid, caffeic acid, catechin, epicatechin, kaempferol-3-O-glucoside, quercetin-3-O-glucoside, cyanidin-3-O-glucoside, delphinidin-3-O-glucoside, malvidin-3-O-glucoside, petunidin 3-glucoside, and peonidin 3-O-glucoside) were purchased from Sigma-Aldrich (St Louis, MO, USA) and were used to optimize Selected Ion Response (SIR) parameters that were used for the identification and quantification of target compounds. Other phenolics identified in the method were quantified by the response curves from the closest phenolic species. Anthocyanin species were designated as 3-O-glucoside by the comparison of chromatography and SIR to authentic standards. The designation of arabinoside and galactoside was performed based on elution order from our previous separations [20,22]. 

### 2.5. Sample Collection and Preparation

A blood sample was collected after a 12 h, overnight fast. Then, a meal tolerance test was conducted, followed by a 2 h post-prandial blood collection. PBMC samples were collected by using cell preparation tubes (BD Vacutainer, Franklin Lakes, NJ, USA). Five 8 mL vials were collected at both time-points and processed following the manufacturer’s instructions. In a sterilized laminar flow hood, the resultant PBMC layer was pooled into a single 50 mL Falcon tube (approximately 30–40 mL) and spiked with DMSO (Sigma-Aldrich, St. Louis, MO, USA) at 10% of the pooled volume (approximately 3–4 mL). The mixture was inverted gently and aliquoted evenly into ten 5 mL screw-top cryovials. The cryovials were placed in Mr. Frosty containers (Nalgene, Rochester, NY, USA) and placed at −80 °C for at least 4 h before transfer into liquid nitrogen dewars for long-term storage. 

On the day of high-resolution respirometry, eight of the ten stored cryovials were thawed in a 37 °C water bath for approximately 2 min. The thawed PBMCs were split evenly between two 50 mL cryovials, and the volume was doubled with HBSS (Sigma-Aldrich, St. Louis, MO, USA). After gentle inversion, the samples were centrifuged for fifteen minutes at 4 °C and 300 RCF. Following a second rinse step in HBSS, the PBMC sample was resuspended in MiR05 media warmed to 37 °C [23]. Cell viability was measured by using a Neubauer Hemocytometer, and all samples were above 94% viable. Sample density ranged from 4 to 13 × 10^6^ viable PBMCs per mL of MiR05 media. 

### 2.6. High-Resolution Respirometry

Oxygen concentration in MiR05 solution was measured by using an oxygraph (Oroboros, Austria) Clark-type electrode, which interfaces with the manufacturer’s software (Datlab, version 6.1) to calculate the rate of oxygen consumption in real time (pmol O2 per second per million cells). The oxygraph is equipped with two independent chambers, and each sample was measured simultaneously in both chambers. PBMC respirometry was characterized by using a modified protocol for permeabilized cells oxidizing a fatty acid substrate, as described previously [24]. Deviations from the referenced protocol include the use of octanoyl-carnitine in place of palmitoyl-carnitine, as preliminary experiments confirmed a stronger signal with the former, and substrate additions were at a concentration that was recommended by the manufacturer to avoid rate-limiting conditions. The nine-step protocol allows for the characterization of cellular activity across different respiration states, and samples were treated with the following substrates in sequence: 10 μg digitonin, 200 μM octanoyl-carnitine, 2 mM malate, 2.5 mM adenosine diphosphate (ADP), 10 μM cytochrome c, 10 mM glutamate, 10 mM succinate, 5 μg oligomycin, 0.5 μM steps of carbonyl cyanide 4-(trifluoromethoxy) phenylhydrazone (FCCP) [25,26].

### 2.7. DNA Extraction and Mitochondrial PCR

Respirometry measurements are commonly corrected by a factor that accounts for mitochondrial density and/or activity. Respirometry measurements that were performed on PBMC samples were corrected for mitochondrial density by using RT-PCR [27,28]. DNA was extracted by using the DNeasy blood and tissue kit (Qiagen, Germantown, MD, USA) according to the manufacturer’s instructions. DNA was quantified by using a nanodrop spectrophotometer (Thermofisher, Wilmington, DE, USA). DNA content was normalized to 3 ng per µL, and 6 ng was added to each PCR reaction in triplicate for each PBMC sample. PrimePCR SYBR custom assay forward and reverse primers for both mitochondrial and nuclear DNA were prepared by Bio-Rad (Hercules, CA, USA) according to previously reported primer sequences [27,28]. Relative mitochondrial content was calculated as described by Venegas et al.: ∆Ct = (nuclear DNA average Ct)—(mitochondrial DNA average Ct) and mitochondrial DNA content = 2 × 2^(∆Ct). 

### 2.8. Calculations and Statistics

Cellular respiration was expressed per million viable cells and was also expressed after correction for mitochondrial content, and statistical tests were performed to determine differences in respiration due to diet treatment across different respiration states. Substrate and coupling control ratios were calculated as described by the manufacturer and also tested for differences due to diet treatment [26]. Briefly, the substrate control ratio (SCR) is the ratio of state 3 to state 4 respiration; it is a measure of the capacity of oxidative phosphorylation on octanoyl-carnitine in a constant coupling state. The coupling control ratio (CCR) is the ratio of uncoupled to state 3 respiration; it is a measure of the electron transport capacity relative to the maximal state of oxidative phosphorylation. The control ratios serve as a marker of internal normalization, i.e., they are independent of mitochondrial density and are meant to assess mitochondrial quality [26]. Reserve capacity is the maximal respiration minus basal respiration. Outcome measures were analyzed by PROC MIXED, by using SAS version 9.4 (SAS institute, Cary, NC, USA), with the subject as a random effect and BMI, sex, age, sequence, period, and treatment as fixed effects. Data are presented as LSmeans for each treatment, and *p* < 0.05 was considered statistically significant.

## 3. Results

**Figure 1:**
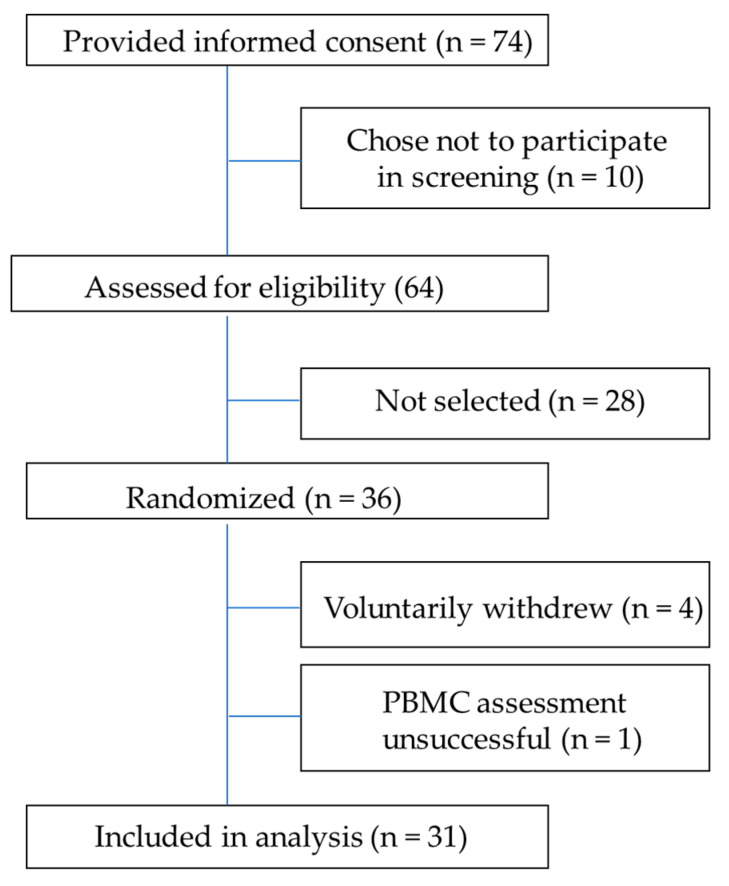
CONSORT (Consolidated Standards of Reporting Trails) diagram for cellular respiration study.

In total, 74 potential volunteers provided informed consent, 64 were screened for eligibility, and 36 were selected for participation in the study. Of the 36 enrolled subjects, 4 subjects withdrew from the study, and 1 subject’s PBMCs were unsuccessfully measured in the oxygraph. PBMCs were successfully processed and assessed from 31 subjects for high-resolution respirometry. The CONSORT diagram is shown in Figure 1. The study included 16 females (6 overweight, 10 obese) and 15 males (8 overweight, 7 obese). Average weight, BMI, and age separated by sex and overweight/obese classification are reported in Table 2. 

The treatment food daily servings were scaled by energy intake. The treatments were matched for calories and sugar/monosaccharide content. Berry treatments provided anthocyanins, flavonols, flavan-3-ols, and phenolic acids (Table 3). Anthocyanin intake ranged from 109 mg/d to 218 mg/d for whole berries (mean intake 143.5 mg/d) and 77 mg/d to 155 mg/d for berry juice (mean intake 101 mg/d). The whole berries also provided mean intakes of 15 mg/d flavonols, 3 mg/d flavan-3-ols, and 38 mg/d phenolic acids, while the berry juice provided mean intakes of 6 mg/d flavonols, 1 mg/d of flavan-3-ols, and 6 mg/d phenolic acids. The berry anthocyanins included glycosides of cyanidin, delphinidin, malvidin, peonidin, and petunidin. The distributions of anthocyanins were different between the whole berries and juice treatments, reflecting differences in the extractability of the anthocyanins from the different berry matrices by pressing. For the whole berries, cyanidin 3-glucoside (56–112 mg/d), malvidin 3-arabinoside (12–24 mg/d), and malvidin 3-galactoside (10–20 mg/d) were present in the largest quantities. For the berry juice, cyanidin 3-glucoside was present in the largest amount (25–50 mg/d), followed by peonidin 3-galactoside (13–26 mg/d), cyanidin 3-galactoside (12–25 mg/d), and peonidin 3-arabinoside (10–19 mg/d). 

In the fasted state, after subjects had consumed a base diet for one week with one of the treatment foods, overall, the anthocyanin-rich berry juice supported the highest rate of oxygen consumption, while the fiber-enriched gelatin was associated with the lowest rate of oxygen consumption. Alternatively, in the fed state after the meal challenge containing one of the treatment foods, the fiber-enriched gelatin treatment supported the highest oxygen consumption by PBMCs. Differences in oxygen consumption were observed when the data were normalized per million viable cells and by mitochondrial DNA. The oxygen consumption measured in these experiments represents cellular respiration rather than solely mitochondrial respiration, though mitochondrial respiration would be expected to account for almost the entirety of the respiration. In the following text, “per 10^6^ cells” refers to oxygen consumption normalized per million viable cells, while “per MtDNA” refers to oxygen consumption normalized by mitochondrial DNA. 

**Figure 2:**
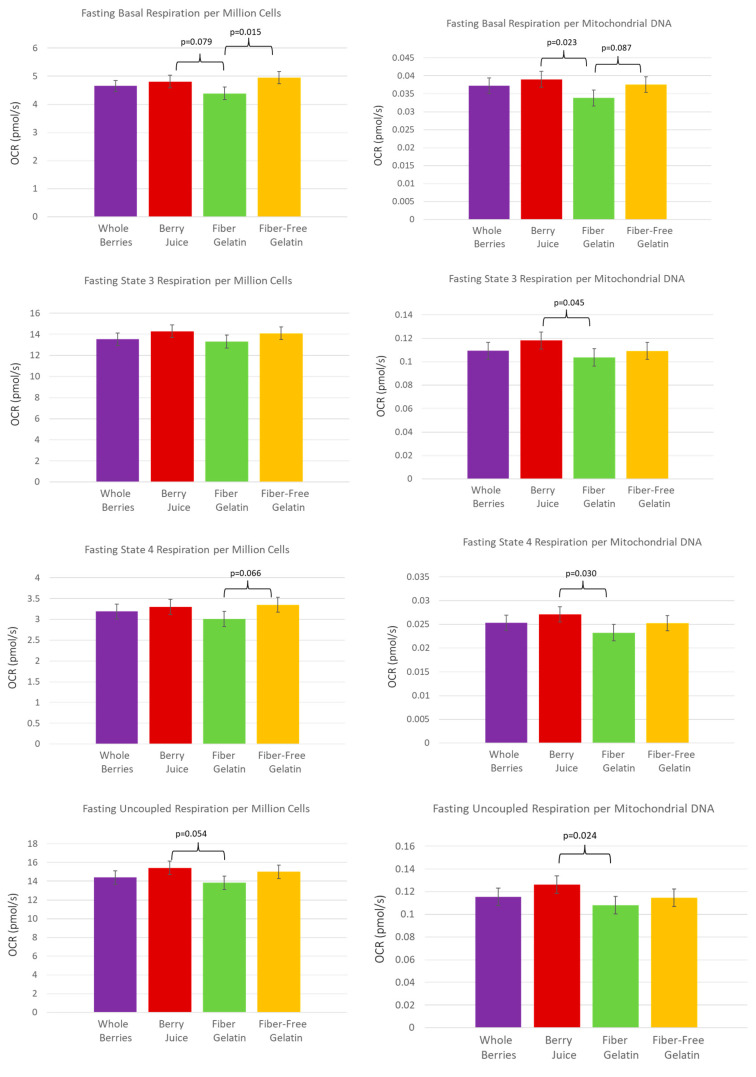
PBMC oxygen-consumption rate in the fasted state after subjects consumed whole berries, berry juice, fiber-enriched gelatin, or fiber-free gelatin for one week.

**Figure 3:**
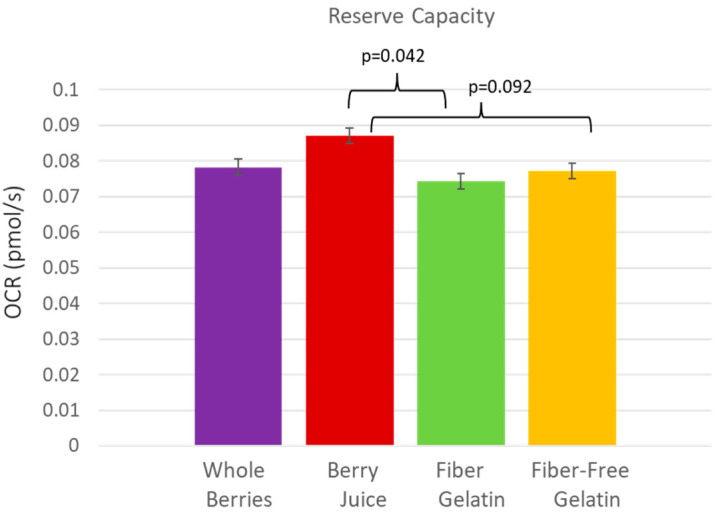
Reserve capacity in PBMCs after subjects consumed whole berries, berry juice, fiber-enriched gelatin, or fiber-free gelatin.

The anthocyanin-rich berry juice treatment supported the highest rate of cellular respiration in the fasted state, after subjects had consumed the berry juice treatment daily for one week. The OCR (oxygen-consumption rate) per MtDNA was significantly higher after a week of berry juice consumption compared to fiber-enriched gelatin for basal respiration (*p* = 0.023), State 3 respiration (*p* = 0.045), State 4 respiration (*p* = 0.030), and uncoupled respiration (*p* = 0.024). Values were also notable for OCR per 10^6^ cells, though they did not quite meet statistical significance, with berry juice OCR exceeding fiber-enriched gelatin for basal respiration (*p* = 0.079) and uncoupled respiration (*p* = 0.054). These comparisons are shown in Figure 2. Reserve capacity was also improved after the consumption of juice. Reserve capacity was higher for juice vs. fiber-enriched gelatin (*p* = 0.042), and reserve capacity values were higher for juice vs. fiber-free gelatin, though these values did not quite meet statistical significance (*p* = 0.092) (Figure 3). 

Fiber-enriched gelatin consumption during the prior week resulted in the lowest cellular respiration rate in the fasted state. OCR by PBMCs after subjects consumed a diet with fiber-enriched gelatin was lower than after the consumption of fiber-free gelatin for basal respiration (per 10^6^ cells, *p* = 0.015) and also tended to be lower when expressed per MtDNA (fiber-enriched gelatin tending toward a lower value than control, *p* = 0.087). In State 4 respiration, OCR per 10^6^ cells tended toward a lower value for fiber-enriched gelatin compared to fiber-free gelatin (*p* = 0.066). These comparisons are shown in Figure 2. 

**Figure 4:**
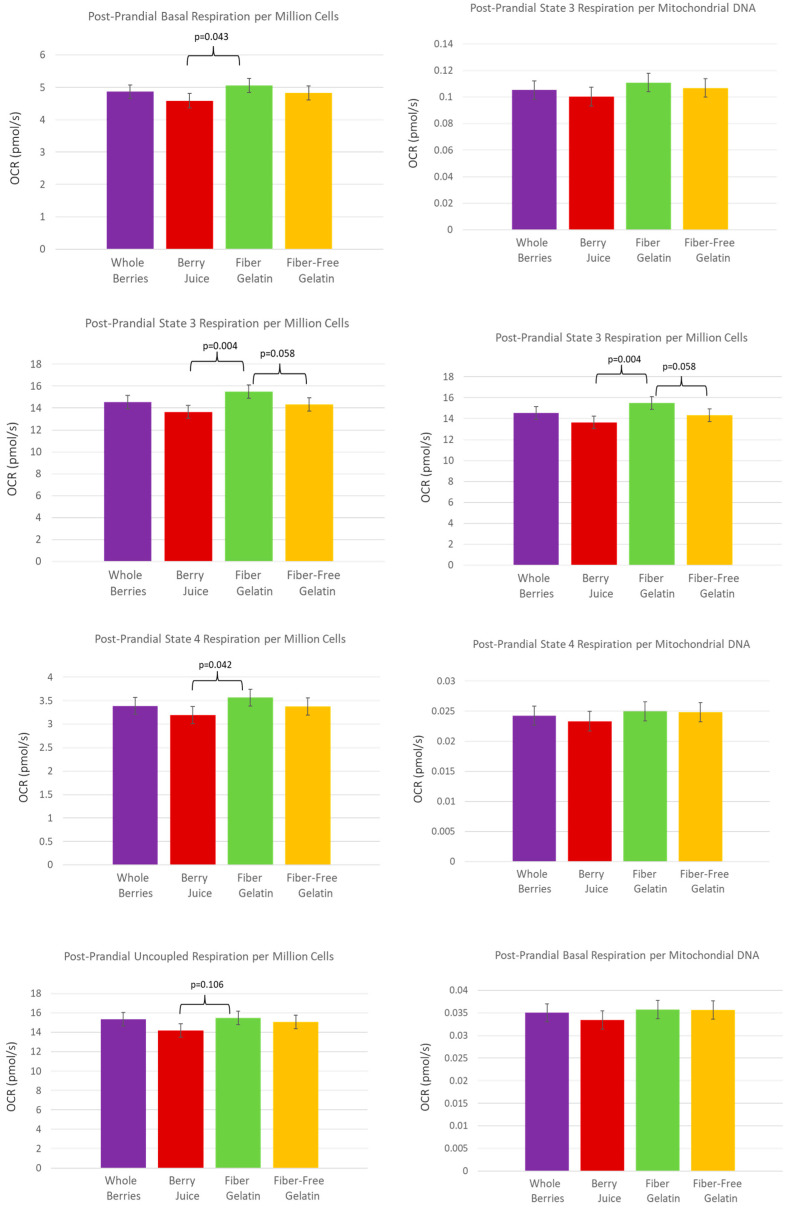
PBMC oxygen-consumption rate in the fed state after subjects consumed a test meal with whole berries, berry juice, fiber-enriched gelatin, or fiber-free gelatin, having also consumed the respective treatment for one week prior to the test meal.

In the fed state, after subjects had consumed a meal with the assigned treatment food, having also consumed the treatment food daily for the previous week, OCR tended to be highest for the fiber-enriched gelatin. Post-prandially, OCR per 10^6^ cells was higher for fiber-enriched gelatin than for berry juice for basal respiration (*p* = 0.043), for State 3 respiration (*p* = 0.004), and for State 4 respiration (*p* = 0.042). These comparisons are shown in Figure 4. The OCR for fiber-enriched gelatin compared to fiber-free gelatin for State 3 respiration was also notable, with the OCR per 10^6^ cells tending to be higher for fiber-enriched gelatin (*p* = 0.058).

## 4. Discussion

The anti-obesity effects of berry and/or anthocyanin consumption have been demonstrated in multiple rodent studies, as reviewed by Riordan and Solverson [1] and Jayarathne et al. [2]. Our earlier work suggested translatability of these berry effects to humans, as we reported increases in fat oxidation [3] and insulin sensitivity [3,19] in overweight or obese individuals consuming berries. Understanding mechanisms of action in vivo is important for extending these benefits to other foods and dietary patterns. 

PBMC use for the investigation of cellular level changes in metabolism in clinical nutrition studies has the advantage of low-risk, minimally invasive collection. Successful collection and processing can be performed in a standard clinical setting without specialized training. Earlier human work describes the utility of PBMCs as a candidate tissue for the study of metabolic changes induced by fasted/fed states, particularly with fatty acid oxidation [29]. In our hands, preliminary experiments assessing the sample storage method described above resulted in no impact on signal quality of the samples used in the respirometry protocol. This validated sample storage technique is particularly advantageous for clinical trials involving numerous blood collections spanning several months; analyzing fresh samples may be logistically difficult, and our approach allowed for dedicated sample analysis after the completion of the human feeding study. To ensure reliable signal strength during respirometry, we recommend a cell density between 5 and 10 × 10^6^ viable PBMCs per mL of respiration medium, or the collection of approximately 32–40 mL of whole blood per sampling if performing respirometry in duplicate, in addition to measuring mitochondrial content.

Blood-based bioenergetic profiling has demonstrated good associations with skeletal and cardiac muscle bioenergetic capacity, reflecting that blood cell respiration is a good model for other tissues [15]. Moreover, PBMC respiration has also been associated with health [12,14]. Tyrell et al. [14] report several associations with uncoupled respiration and measures of physical function and strength in geriatric, overweight, and obese adults. In a group of male and female subjects aged 65 years and older, higher uncoupled respiration was associated with greater knee extensor and grip strength and lower plasma interleukin-6 concentrations. Higher spare respiratory capacity (reserve capacity) was also associated with higher scores of the outcomes listed above, in addition to higher scores on the expanded short physical performance battery (a combination gait speed test, repeated chair stands, and balance tests) and greater leg muscle quality (knee extensor torque divided by leg lean mass). An earlier study by the same group reported positive associations of the respirometry profile of both PBMC and leg muscle biopsies with gait speed in older, overweight, and obese subjects [12].

In the present study, the berry juice had a greater impact on fasting cellular respiration than the whole berries, even though the whole berries had a higher content of phenolic compounds compared to the juice. One possible explanation is that the bioavailability of the compounds from the juice was greater than that from berries. Alternatively, the distribution of phenolic compounds in the juice and the whole berries was slightly different, which could have impacted biological activity. Finally, the whole berries contained fiber and other components that were absent from the juice, and those other components may have also impacted the results. 

Previous in vitro studies have demonstrated that anthocyanin-rich treatments affect mitochondrial function, which represents the primary source of cellular respiration. Giampieri et al. [30] exposed human dermal fibroblasts to strawberry extract. The strawberry extract improved both basal oxygen consumption and maximal respiratory capacity. De Sales et al. [31] demonstrated a 30% increase in routine respiration and a 50% increase in coupled respiration in HepG2 cells exposed to anthocyanin-rich grape pomace extract. In another study, 3T3-L1 adipocytes increased maximal respiration when incubated with malvidin 3-glucoside [5]. In addition, our group recently found a greater oxidative capacity of 3T3-L1 adipocytes treated with cyanidin 3-glucoside or blackberry extract [32]. Oxygen consumption was higher in all respiration states.

There are multiple molecular mechanisms by which anthocyanins and/or their metabolites may affect mitochondrial function. In a study of mice that were fed bilberry and black currant, liver homogenates showed increased expression of mitochondrial electron transport complexes 1 and 4 [33]. Skemiene et al. [34] studied the effect of five anthocyanins on extra-mitochondrial cytochrome c in vitro. Both delphidin 3-glucoside and cyanidin 3-glucoside were potent reducers of cytochrome c. In a study of isolated mitochondria, the use of fluorescence probes showed that an anthocyanin-rich extract supplied compounds that could be incorporated into the mitochondrial membrane and cross the membrane into the inner mitochondrial space [35]. Their results suggested that compounds in the anthocyanin-rich extract could interact with components in the redox chain to influence pathways of electron transport.

The metabolites of the gut microbiota may also have impacts on cellular respiration or mitochondrial function. Berry components can serve as substrates for metabolism by gut microbiota to produce other bioactive compounds. For example, blackberries and strawberries are rich sources of ellagitannins and ellagic acid, which are ultimately metabolized to urolithins by gut microbes. Urolithin A has multiple effects leading to enhanced mitochondrial function [36]. However, the current evidence suggests that only about 40% of individuals seem to harbor microbiota capable of producing significant amounts of urolithin A [37].

The high-fiber treatment increased oxygen consumption in the fed state, but not in the fasted state. One possible explanation is increased circulating short-chain fatty acids (SCFAs) after the meal, not from the challenge meal, but from previous meals. The subjects consumed the high-fiber gelatin treatment for one week prior to the meal challenge. Therefore, the gut microbiota had been chronically exposed to substrate for fermentation to SCFAs. The fiber source was cellulose, and while early studies of microcrystalline cellulose suggested that only a limited number of individuals have significant amounts of cellulolytic gut microbes [38], studies since that time have demonstrated that the majority of individuals do actually harbor gut microbiota capable of fermenting cellulose [39]. Cellulose fermentability in humans fed mixed plant sources (such as those used in the present study) ranges from 42% to 80% [40,41,42,43]. Previous studies of daily fiber intake have demonstrated that SCFAs in blood do not increase significantly in the fasted state but do increase post-prandially, presumably from previous fiber-rich meals [44,45]. Lappi et al. [45] observed no increase in SCFAs in fasting plasma after daily consumption of high-fiber rye bread for 4 weeks compared to refined wheat bread. However, they observed a rapid post-prandial rise in plasma butyrate or propionate after a standardized challenge meal when subjects had consumed a high-fiber diet daily prior to the challenge meal, and they presumed that the SCFAs were derived from fiber in previous meals. Other studies have also demonstrated the delayed appearance of SFCAs after a fiber-rich meal [46,47]. Priebe et al. [46] demonstrated a greater post-prandial appearance of plasma butyrate when the previous meal was high in fiber compared to a low-fiber control. Similarly, Nilsson et al. [47] reported that a high-fiber evening meal increased post-prandial plasma butyrate the next morning. SCFAs function as an energy source [48,49] and supply reducing equivalents to the electron transport chain. Furthermore, in a study of overweight/obese men, colonic SCFA infusions increased fat oxidation, energy expenditure, and PYY [49].

## 5. Conclusions

In conclusion, these results confirm the influence of berry treatments on bioenergetics in humans. The effects appear to be different for different berry components, and effects differ between the fasted and fed state. Of the anthocyanin-/flavonoid-rich treatments, berry juice conveyed the greatest increase in respiration, which may have been the result of the improved bioavailability of compounds in juiced or processed berries. 

## Figures and Tables

**Table 1:** Mean daily intake for treatment foods and components ^1^.

	Gelatin Control	Whole Berries	Berry Juice	Gelatin + Fiber
Daily dose (g)	523	523	502	523
Total sugar (g)	37.6	37.6	37.4	37.6
Glucose (g)	19.6	19.6	19.6	19.6
Fructose (g)	18.0	18.0	17.8	18.0
Soluble fiber (g)	0	0	0	0
Insoluble fiber (g)	0	9.5	0	9.5
Anthocyanins (mg)	0	143.5	101.1	0
Flavonols (mg)	0	15.3	6.4	0
Flavan-3-ols (mg)	0	2.9	0.7	0
Phenolic acids (mg)	0	38.2	6.1	0

^1^ Treatment doses were scaled according to total energy intake for each subject; values are mean daily intakes.

**Table 2:** Subject characteristics at baseline ^1^.

	Females (n = 16)		Males (n = 15)	
	Overweight (n = 6)	Obese (n = 10)	Overweight (n = 8)	Obese (n = 7)
Weight, kg	67.9 ± 3.1	89.8 ± 10.6	82.7 ± 11.0	100.5 ± 8.0
BMI, kg/m^2^	26.6 ± 0.7	33.7 ± 2.9	26.9 ± 1.3	32.2 ± 1.3
Age, y	59.3 ± 9.4	60.2 ± 8.1	60.0 ± 13.3	62.1 ± 10.3

^1^ Values are means ± SDs.

**Table 3:** Phenolic compounds in berry treatments ^1^.

	mg per 100 g Fresh Weight	
	Whole Berries	Berry Juice
Anthocyanins		
Cyanidin-3-Gal	1.70 ± 0.08	3.19 ± 0.05
Cyanidin-3-Glu	14.05 ± 0.96	6.57 ± 0.20
Cyanidin-3-Ara	0.906 ± 0.168	2.01 ± 0.05
Delphinidin-3-Gal	0.874 ± 0.103	0.101 ± 0.002
Delphinidin-3-Glu	ND	trace
Delphinidin-3-Ara	0.830 ± 0.081	0.053 ± 0.002
Malvidin-3-Gal	2.54 ± 0.15	1.05 ± 0.06
Malvidin-3-Glu	0.500 ± 0.060	0.012 ± 0.003
Malvidin-3-Ara	3.05 ± 0.17	0.805 ± 0.017
Peonidin-3-Gal	0.793 ± 0.102	3.43 ± 0.10
Peonidin-3-Glu	0.070 ± 0.012	0.17 ± 0.02
Peonidin-3-Ara	0.588 ± 0.075	2.53 ± 0.06
Petunidin-3-Gal	0.920 ± 0.039	0.159 ± 0.003
Petunidin-3-Glu	0.340 ± 0.011	0.016 ± 0.004
Petunidin-3-Ara	0.076 ± 0.066	0.057 ± 0.002
Total Anthocyanins	27.2 ± 1.9	20.1 ± 0.4
Flavan-3-ols		
Catechin	0.226 ± 0.026	0.079 ± 0.003
Epicatechin	0.330 ± 0.016	0.039 ± 0.001
Total Flavan-3-ols	0.556 ± 0.041	0.118 ± 0.004
Flavonols		
Quercetin-3-Gal	2.01 ± 0.64	1.00 ± 0.01
Quercetin-3-Glu	0.448 ± 0.001	0.088 ± 0.001
Kaempferol-3-Glu	0.466 ± 0.042	0.186 ± 0.003
Total Flavonols	2.93 ± 0.67	1.27 ± 0.01
Phenolic Acids		
Chlorogenic acid	7.09 ± 0.27	1.08 ± 0.02
Caffeic acid	0.211 ± 0.013	0.032 ± 0.002
Gallic acid	ND	0.116 ± 0.004
Total Phenolic acids	7.30 ± 0.28	1.23 ± 0.02

^1^ Values are mean + SD; ND indicates not detected.

## Data Availability

Only completely de-identified data will be made available. Requests for data should be directed to the corresponding author.
